# Synergisms of Microbial Consortia, N Forms, and Micronutrients Alleviate Oxidative Damage and Stimulate Hormonal Cold Stress Adaptations in Maize

**DOI:** 10.3389/fpls.2020.00396

**Published:** 2020-04-24

**Authors:** Narges Moradtalab, Aneesh Ahmed, Joerg Geistlinger, Frank Walker, Birgit Höglinger, Uwe Ludewig, Günter Neumann

**Affiliations:** ^1^Institute of Crop Science (340h), University of Hohenheim, Stuttgart, Germany; ^2^Institute of Bioanalytical Sciences, Anhalt University of Applied Sciences, Bernburg, Germany; ^3^Institute of Phytomedicine (360), University of Hohenheim, Stuttgart, Germany

**Keywords:** synergisms, plant growth-promoting microorganisms, ammonium, colding, phytohormones, ROS detoxification

## Abstract

**Aims:**

Low soil temperature in spring is a major constraint for the cultivation of tropical crops in temperate climates. This study aims at the exploitation of synergistic interactions of micronutrients, consortia of plant growth-promoting microorganisms and N forms as cold-stress protectants.

**Methods:**

Maize seedlings were exposed for two weeks to low root zone temperatures at 8–14°C under controlled conditions on a silty clay-loam soil (pH 6.9) collected from a maize field cultivation site. A pre-selection trial with fungal and bacterial PGPM strains revealed superior cold-protective performance for a microbial consortium of *Trichoderma harzianum* OMG16 and *Bacillus spp.* with Zn/Mn supplementation (CombiA^+^), particularly in combination with N-ammonium as a starting point for the characterization of the underlying physiological and molecular mechanisms.

**Results:**

In nitrate-treated plants, the cold stress treatment increased oxidative leaf damage by 133% and reduced the shoot biomass by 25%, related with reduced acquisition of phosphate (P), zinc (Zn) and manganese (Mn). The supplying of N as ammonium improved the Zn and Mn nutritional status and increased the ABA shoot concentration by 33%, as well as moderately increased detoxification of reactive oxygen species (ROS). Moreover, use of N as ammonium also increased the root auxin (IAA) concentration (+76%), with increased expression of auxin-responsive genes, involved in IAA synthesis (*ZmTSA*), transport (*ZmPIN1a*), and perception (*ZmARF12*). Additional inoculation with the microbial consortium promoted root colonization with the inoculant strain *T. harzianum* OMG16 in combination with ammonium fertilization (+140%). An increased ABA/cytokinin ratio and increased concentrations of jasmonic (JA) and salicylic acids (SA) were related to a further increase in enzymatic and non-enzymatic ROS detoxification. Additional supplementation with Zn and Mn further increased shoot IAA, root length and total antioxidants, resulting in the highest shoot biomass production and the lowest leaf damage by oxidative chemical species.

**Conclusion:**

Our results suggest the mitigation of cold stress and reduction of stress priming effects on maize plants due to improved ROS detoxification and induction of hormonal stress adaptations relying on the strategic combination of stress-protective nutrients with selected microbial inoculants.

## Introduction

The cultivation of tropical and subtropical crops in agricultural production systems under temperate climates continuously increases and is further promoted by global warming. Under these conditions, short vegetation periods due to low temperatures in early spring remain a major challenge for crops, such as maize, tolerating soil temperatures not much lower than 15°C for normal germination and early growth (Cutforth et al., 1986; Kaspar and Bland, 1992). This is further complicated by the more widespread adoption of no-tillage or conservation tillage, leading to a slower seedbed warming in spring (Hayhoe et al., 1996).

As a consequence of cold stress (5−15°C), poor field establishment due to inhibition of root development, impaired uptake and translocation of water and nutrients can translate into poor vegetative growth, low-stress resistance and finally reduction of yield (Duncan and Hesketh, 1968; Muldoon et al., 1984; Imran et al., 2013). Besides, the maize shoot meristem is directly affected since it remains belowground even until the V6 stage (Stone et al., 1999). Impairment of root growth particularly limits the acquisition of phosphate (P) and micronutrients, such as zinc (Zn), manganese (Mn) and iron (Fe) due to low soil mobility, leading to induced nutrient deficiencies (Engels and Marschner, 1996; Bradacova et al., 2016; Moradtalab et al., 2018). Due to the importance of micronutrients as co-factors for enzymatic and non-enzymatic detoxification of reactive oxygen species (ROS), oxidative stress appears in consequence of ROS overproduction, which causes a severe damage to membranes, organelles, and cell functions (Cakmak, 2000; Gong et al., 2005). Impairment of photosynthesis due to oxidative leaf damage and impaired auxin production related to zinc limitation are factors further contributing to inhibition of root growth, impaired nutrient acquisition and limited plant regrowth (Moradtalab et al., 2018).

Natural cold stress adaptations are weakly expressed in tropical and subtropical-originated plant species. Most breeding programs toward improved cold tolerance use Flint maize inbred lines as a source of adaptation, originally based on the Northern Flint race adapted to cold temperate regions of Northeastern America (Riva-Roveda et al., 2016). Temporary growth and rapid recovery from limitations due to low photosynthesis rates have been described as major adaptation traits of these genotypes (Riva-Roveda et al., 2016). As a complementary approach, adapted fertilization for supplementation of critical nutrients, such as phosphate (P) and micronutrients or application of stress-protective biostimulants are discussed as mitigation strategies to cope with cold-stress (Bradacova et al., 2016; Gómez-Muñoz et al., 2018; Moradtalab et al., 2018):

(i) Placement of P starter fertilizers as ammonium phosphates close to the seed is meanwhile regarded as a standard measure for maize cultivation in temperate climates (Nkebiwe et al., 2016). Both, P and ammonium N are applied close to the seedling root, where rhizosphere acidification, induced by preferential ammonium uptake (Marschner and Römheld, 1983) can increase the solubility of critical nutrients, such as P, Zn Mn, Fe, and Cu, with particular importance on soils with neutral to alkaline pH (Neumann and Römheld, 2002; Jing et al., 2010).
(ii) The application of stress-protective nutrients, such as Zn, Mn, Fe, B, Cu, or Si by seed treatments or starter fertigation to promote oxidative stress defense, is another strategy with proven beneficial effects on cold tolerance in maize (Imran et al., 2013; Bradacova et al., 2016; Moradtalab et al., 2018).
(iii) Also, inoculation with plant growth-promoting microorganisms (PGPMs) is discussed as a potential measure to promote early growth and field establishment of sensitive crops under challenging environmental conditions (Kumar and Verma, 2018). In the context of cold tolerance, the selection of so-called psychrotolerant PGPM strains with the ability to propagate also at soil temperatures below 15°C and sometimes even close to the freezing point, may provide a significant advantage (Selvakumar et al., 2008a, b; Subramanian, 2011). The same holds true for the use of microbial consortia as plant inoculants, combining different PGPM strains with complementary properties and differing stress tolerance (Nuti and Giovannetti, 2015; Woo and Pepe, 2018). A common feature of the described mitigation strategies is the mode of application of the aforementioned agricultural inputs. This may offer opportunities for the development of multifunctional products, combining beneficial properties and exploiting potential synergisms.

Based on this hypothesis, the aim of this study was to explore the synergistic interactions and the combined application of PGPMs, micronutrients (Zn and Mn), and the use of N as ammonium and nitrate on the recovery and early growth of maize after two-weeks exposure to low root zone temperatures at 8–14°C on a silty clay-loam field soil, collected from a maize cultivation site.

A pre-selection trial was conducted with a range fungal and bacterial PGPM strains based on *Penicillium sp.* with cold-protective properties (Gómez-Muñoz et al., 2018), a cold-tolerant strain of *Bacillus atrophaeus* (ABI02) and a microbial consortium product (MCP), based on a combined formulation of *Trichoderma harzianum* OMG16 and *Bacillus spp.* with Zn/Mn supplementation (CombiA). The CombiA consortium was selected according to the MCP concept, by combining different as microbial strains with complementary properties as discussed an approach to increase the efficiency and the flexibility of PGPM-based production strategies under variable environmental conditions (Woo and Pepe, 2018). *Trichoderma-Bacillus* combinations are among the well-documented examples in this context. Although co-cultivation of *Trichoderna* and *Bacillus* strains on artificial growth media was frequently characterized by antagonisms (Gyu Kim et al., 2008), in many plant species, including *Oryza sativa* (Ali and Nadarajah, 2014), *Triticum aestivum* (Karuppiah et al., 2019), *Cicer arietinum* (Zaim et al., 2018), *Solanum melongena* and *Capsicum annuum* (Abeysinghe, 2009), synergistic beneficial effects were reported after co-inoculation. This included stimulation of germination and growth promotion, as well as biocontrol effects against fungal pathogens, such as *Rhizoctonia, Fusarium* and *Pythium*, known as important damping-off diseases in cold and wet soils with potential relevance also for the present study. Testing seven different fungal and bacterial inoculants, Mpanga et al. (2019b) found superior root growth-promoting properties and improved nutrient acquisition after CombiA inoculation in maize under P limitation. Similarly, superior cold-protective performance in terms of biomass production and reduction of oxidative leaf damage was reported for the CombiA consortium in the previous selection trial conducted in this study (Supplementary Table S1).

To evaluate the mode of action of the selected cold stress mitigation strategies, we hypothesized that (i) ammonium-dominated N supplying will increase the availability of critical nutrients, such as P, Zn, Mn, Fe via rhizosphere acidification on the investigated soil with neutral pH and additionally stimulate rhizosphere interactions with the selected inoculants as previously reported by Mpanga et al. (2019a), (ii) The starter application of micronutrients will additionally provide co-factors (Zn, Mn) for the systems of ROS detoxification already before the onset of the cold stress treatments. The improved micronutrient status will support the expression of adaptive responses to cold stress by mitigation of oxidative damage, as similarly reported by Bradacova et al. (2016) and Moradtalab et al. (2018), (iii) The selected microbial inoculants will interact with plant hormonal homeostasis, supporting nutrient acquisition by stimulation of root growth, induction of stress priming effects and provide protection against pathogen attack.

To dissect the investigated mitigation strategies to low temperatures, the monitoring of physiological stress indicators, hormonal homeostasis and expression of related genes was conducted for the single and combined application of the selected cold-stress protectants.

## Materials and Methods

### Plant Cultivation

For all experiments, maize (*Zea mays* L. cv. Rolandinio) plants were grown for six weeks under greenhouse conditions. For the preparation of the growth substrate, a silty clay loam field soil pH 6.9 (see Table 1 for soil properties) was freshly collected from the Ap horizon of a maize cultivation site at the Hohenheim University experimental station Ihinger Hof (48°45’ N, 8°56’ E, Renningen, Germany), air-dried and sieved using 2 mm mesh size. The soil substrate was mixed with quartz sand (30% w/w) to improve the soil structure. Fertilization was performed with Ca(H_2_PO_4_)_2_: 80 mg kg^–1^ dry matter (DM) P; K_2_SO_4_: 150 mg kg^–1^ DM K and MgSO_4_: 50 mg kg^–1^ DM Mg. Nitrogen was applied at a rate of 100 mg kg^–1^ DM N, either as a commercial Ca-nitrate formulation, (YaraLiva^®^ CALCINIT^®^, YARA GmbH & Co., KG, Germany) or as stabilized ammonium sulfate (NovaTec^®^ Solub 21, COMPO EXPERT GmbH, Germany).

**TABLE 1:** Chemical and physicochemical properties of the soil sampled at the Hohenheim University experimental station Ihinger Hof Renningen, Germany), 2016.

Determination	Unit	Value
pH-Value (CaCl_2_)		6.9
Available P CAL-Extract VDLUFA)	mg/kg	82.9
Availabke K CAL-Extract VDLUFA)	mg/kg	141.1
Mg (CaCl_2_)	mg/kg	190
Humus	mg/kg	22000
Carbon _total_	mg/kg	12800
N	%	0.177
S	%	0.054
Sand (63–2000 μm)	%	2.9
Silt (2–63 μm)	%	66.8
Clay (<2μm)	%	30.3
Carbonate (Scheibler)	mg/kg	1.1
Fe (CAT-Extract)	mg/kg	126
Cu (CAT-Extract)	mg/kg	4.22
Mg (CAT-Extract)	mg/kg	215
Mn (CAT-Extract)	mg/kg	404
Zn (CAT-Extract)	mg/kg	2.92
K (CAT-Extract)	mg/kg	97.2
P (Olsen)	mg/kg	49.2
B (ICP-OES KW)	mg/kg	17.8
Ca (ICP-OES KW)	mg/kg	4.600
Fe (ICP-OES KW)	mg/kg	26.912
K (ICP-OES KW)	mg/kg	4.407
Cu (ICP-OES KW)	mg/kg	23.1
Mg (ICP-OES KW)	mg/kg	5.630
Mn (ICP-OES KW)	mg/kg	991
P (ICP-OES KW)	mg/kg	990
Zn (ICP-OES KW)	mg/kg	62.6
Base saturation (Co-hexamine VDLUFA)	%	80
Calcium (Ca) exchangeable (CoHexamin)	cmol(c)/kg	13.7
KAK pot (co-hexamine VDLUFA)	cmol(c)/kg	20.6
Potassium (K) exchangeable (CoHexamin)	cmol(c)/kg	0.42
Magnesium (Mg) exchangeable (CoHexamin)	cmol(c)/kg	2.37
Sodium (Na) exchangeable (CoHexamin)	cmol(c)/kg	0

Thereafter, 2 L plastic pots were filled with 1.8 kg of the substrate prior to sowing, which was conducted with five seeds per pot and reduction to one seedling after germination. During the first two weeks, plants were grown under ambient greenhouse temperature conditions (ranging from 18−25°C) and as soon as the plants were established, they were exposed to low root zone temperature (RZT in a thermostatic root-cooling device, described by Bradacova et al. (2016). The root-cooling system is based on an immersion water bath circulator (Thermomix 1480, Frigomix 1497, Braun, Melsungen, Germany) connected to a pipe system, installed in moist peat culture substrate to circulate the cooling fluid through the moist peat layer. For adjustment of the reduced RZT regime (two weeks-cold stress at 8−14°C), the culture vessels were immersed into the cooled peat bed followed by 2-weeks recovery at ambient greenhouse temperature. Soil and air temperatures were constantly recorded by a LogTag device (Supplementary Figure S1). Every second day, soil moisture was adjusted gravimetrically to 70% of the substrate water-holding capacity (18.3% w/w).

### Microbial Inoculants

To select the most effective cold stress-protectants, five different treatments were tested in a pre-screening experiment: (i) Seed dressing with a commercial Zn/Mn formulation (0.2 ml Lebosol^®^ Mn500 SC and 0.1 ml Lebosol^®^ Zn700 SC 100 g^–1^ seeds, Lebosol^®^ Dünger GmbH, Ermstein, Germany); (ii) fertigation with 10^9^ spores Kg^–1^ DM ABI02, a cold-tolerant *Bacillus atrophaeus* strain (ABITEP, Berlin, Germany) combined with Zn/Mn seed dressing, (iii) fertigation with 10^8^ spores Kg^–1^ DM Biological fertilizer OD (BFOD), a *Penicillium* sp. PK112 formulation (Bayer Crop Science Biologicals GmbH, Malchow, Germany) with documented cold−stress protecting effects combined with Zn/Mn seed dressing, (iv) Fertigation with 2.5 × 10^7^ cfu Kg^–1^ DM Combi A^+^, a microbial consortium formulation with Zn (13% w/w) + Mn (9% w/w) + *Trichoderma harzianum* OMG16 (9 × 10^9^ spores g^–1^) + Vitabac (1 × 10^11^ cfu g^–1^, as a mixture of *Bacillus licheniformis, B. megaterium, B. polymyxa, B. pumilis*, and *B. subtilis*, Bactvita GmbH, Straelen, Germany). Untreated variants exposed to cold stress and ambient greenhouse temperature were included as controls. In the follow-up experiments, only CombiA with (CombiA^+^) and without Zn/Mn supplementation (CombiA^–^) were used as inoculants.

The first inoculation was performed one week after sowing (WAS), followed by a 2nd application one day prior to exposure to low RZT 13 days after sowing (DAS) and a final application at the start of recovery phase (29 DAS). All microbial inoculants were applied through soil drenching with a dispenser pipette into the top-soil close to the seedlings roots.

### Assessment of Leaf Damage, Biomass, Root Length, and Rhizosphere pH

At the final harvest (6 WAS), cold stress-induced oxidative leaf damage (chlorosis/necrosis, anthocyanin formation) was quantified by counting the number of damaged leaves plant^–1^. Root systems were excavated, and rhizosphere soil was collected by shaking-off root adhering soil and mixed in a plastic bag. Thereafter, pH was determined according to VDLUFA (1991). Fresh weight (FW) of the shoot and root tissue was measured and finally dry weight (DW) was recorded after oven-drying at 65°C. For root length determination, washed roots previously stored in 30% ethanol, were separated, submerged in a water film on transparent Perspex trays, and subsequently digitalized using a flat-bed scanner (Epson Expression 1000 XL, Tokyo, Japan). The root length of the digitalized samples was measured by the use of the WinRHIZO root analysis system (Reagent Instruments, Quebec, QC, Canada).

### Analysis of Shoot Nutrient Contents

To assess the plant mineral nutrient status, dried shoot material was homogenized using a grinding mill (Labor Scheibenschwingmühle TS-100A, Sieb Technik GmbH, Mühlheim-Ruhr, Germany). After grinding, 250 mg of plant material was ashed in a muffle furnace for five hours at 500°C. After cooling to room temperature, the samples were extracted as described by Moradtalab et al. (2018). Spectrophotometrical determination (Hitachi U-3300, Hitachi Ltd., Corporation Japan) of orthophosphate was conducted by molybdo-vandate method of Gericke and Kurmis (1952). Potassium and Ca were determined by flame emission photometry (ELEX 6361, Eppendorf, Hamburg, Germany). Magnesium, Fe, Mn, Zn, and Cu concentrations were measured by atomic absorption spectrometry (ATI Unicam Solaar 939, Thermo Electron, Waltham, United States).

### Superoxide Dismutase and Peroxidase Activity

Extraction and determination of superoxide dismutase (SOD, EC 1.15.1.1) and peroxidase (POD, EC1.11.1.7) activities were optimized for root and shoot tissues of maize according to the method described (Moradtalab et al., 2018). Spectrophotometrical determination (Spectrophotometer U-3300, Hitachi, Tokyo, Japan) of SOD activity was performed based on the nitro-blue tetrazolium (NBT) method at a wavelength of 650 nm (Beauchamp and Fridovich, 1971). The activity of POD was determined at 470 nm using the tetra-guaiacol assay described by Hajiboland and Hasani (2007).

### Analysis of Metabolites and Antioxidants

Details for metabolite determinations have been described previously by Moradtalab et al. (2018). For the determination of soluble sugars, leaf and root samples were homogenized in 100 mM phosphate buffer (pH 7.5) at 4°C. After centrifugation at 12000 × *g* for 15 min, the supernatant was used for the determination of total soluble sugars by the anthrone-sulfuric acid method of Yemm and Willis (1954). Total phenolics concentration was determined spectrophotometrically at 750 nm, using the Folin method (Hajiboland et al., 2017). For proline analysis, samples were homogenized with 3% (v/v) sulfosalicylic acid and the homogenate was centrifuged at 3,000 × *g* for 20 min. Proline was determined spectrophotometrically at 520 nm after acetic acid and acid ninhydrin derivatization (Bates et al., 1973). The 1,1-diphenyl-2-picrylhydrazyl radical (DPPH) method was used to evaluate the free radical scavenging activity of antioxidants in the plant tissue (Panico et al., 2009).

### UHPLC-MS Analysis of Phytohormones

Frozen maize tissue samples (shoot, roots) of 1 g were ground to a fine powder with liquid nitrogen and extracted twice with 2.5 ml of 80% methanol in falcon tubes. Thereafter, the samples were further homogenized by ultrasonication (Micra D-9 homogenizer, Art, Müllheim Germany) for 1 min and 15 s at 10,000 rpm. Two milliliters of the methanol extracts were transferred to microtubes and centrifuged at 5,645 × *g* for 5 min. Thereafter, 350 μl of the supernatant was mixed with 700 μl ultra-pure water and centrifuged at 5,645 × *g* for 5 min. The supernatant was cleaned through a filtration membrane (Chromafil R O-20/15MS) and transferred to HPLC vials. UHPLC-MS analysis was carried out on a Velos LTQSystem (Thermo Fisher Scientific, Waltham, MA, United States) fitted with a Synergi Polar column, 4 μ, 150 × 3.0 mm, (Phenomenex, Torrance, CA, United States). The injection volume was 3 μL and the flow rate was adjusted to 0.5 ml min^–1^ for gradient elution with mobile phase (A): water and 5% acetonitrile; mobile phase (B): acetonitrile and a gradient profile of 0–1 min, 95% A, 5% B, 11–13 min, 10% A, 90% B, 13.1 min, 95% A, 5% B, 16 min 95% A, 5% B). All standards were purchased from Sigma Aldrich, (Sigma Aldrich, St. Louis, MO, United States) including (±)-jasmonic acid; 3-indoleacetic-acid, gibberellic acid, (±) abscisic acid; *trans-*zeatin; salicylic acid (Moradtalab et al., 2018).

### Expression of Hormonal Target Genes

The expression of selected target genes was analyzed to evaluate potential interactions of applied cold stress protectants with hormonal signaling. Relative expression of the IAA efflux transporter *ZmPIN1a (PINFORMED)*, the auxin response factor 12 (*ZmAFR12*), tryptophan synthase (*ZmTSA*), the auxin early response gene *ZmAuxIAA*, the abscisic acid responsive element-binding factor 2 (*ZmABF2*) and the isopentenyl transferases (*ZmIPT4* and *ZmIPT5*) were determined. Elongation factor-1alpha (*EF1*α) and *tubulin* were selected as reference genes. The results are expressed according to *EF1*α with particularly stable expression under cold stress (Tang et al., 2017). Isolation of mRNA and RT-qPCR quantification of relative transcript abundances was performed from frozen root material by GenXPro GmbH, Frankfurt am Main, Germany.

### Rhizosphere Tracing of *Trichoderma harzianum* OMG16

For the quantification of the *T. harzianum* strain OMG16 (DSMZ accession no.: 32722) in the maize root endosphere, roots were thoroughly cleaned with a soft brush and water to remove residual soil particles, shortly dried between paper towels and cut into small pieces. Approximately 80 mg fine roots were placed in 2 mL tubes containing 1.0 mm silica spheres including one single 0.25-inch ceramic bead (MP Biomedicals, France) and 400 μL peqGOLD lysis buffer (VWR Peqlab, Germany). Root tissue was homogenized for 3 × 30 s at a speed of 6 m/s in a FastPrep 24 bead-beating system (MP Biomedicals). After each cycle samples were cooled on ice for 1 min. DNA was subsequently extracted utilizing the peqGOLD Fungal DNA Kit (VWR Peqlab), following the manufacturer’s instructions. DNA was eluted in a TE buffer (pH 8.0) and checked on 0.8% TAE agarose gels. DNA concentrations were determined using a Qubit^®^ 3.0 Fluorometer and the Qubit dsDNA HS Assay Kit according to the instructions of the manufacturer (Thermo Fisher Scientific, Germany). A *T. harzianum* OMG16-specific primer pair, designed from OMG16 genomic DNA sequences were used for qPCR quantification of *T. harzianum* OMG16 DNA in the DNA samples according to the method described by Mpanga et al. (2019a).

### Statistical Analysis

The study was carried out in a completely randomized design. Data are presented as means ± SD. For statistical analysis of significant differences between treatment groups, a one-way ANOVA followed by a Tukey-test (*p* < 0.05) was performed using the SAS software 9.4 (SAS Institute Inc., Cary, NC, United States). Pairwise comparisons (*t*-Test, *p* < 0.05) were conducted with the Sigma Plot 13 software package (SYSTAT Software Inc., Erkrath, Germany).

## Results

### Cold-Protective Effects of the CombiA + Consortium as Related to N Forms

**FIGURE 1:**
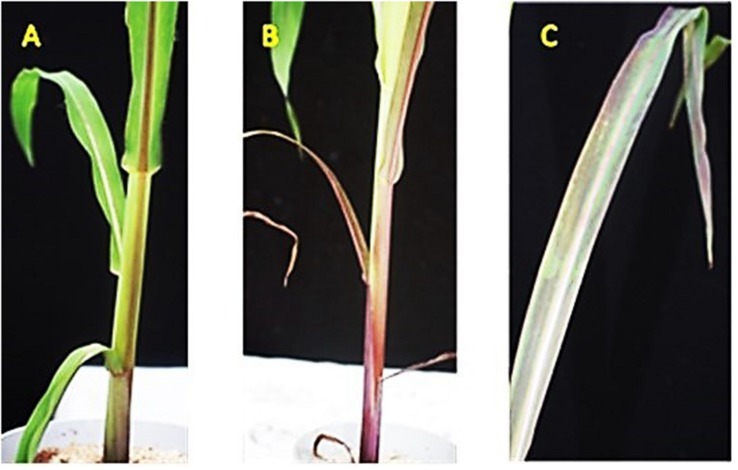
**A** Undamaged leaves of non-stressed maize plants grown for four weeks under ambient greenhouse temperature (18–25°C), **(B,C)** Oxidative leaf damage and symptoms of P limitation (chlorosis, necrosis, stress-anthocyanins) of cold stressed- plants exposed to two weeks of reduced root zone temperature (8–14°C).

**FIGURE 2:**
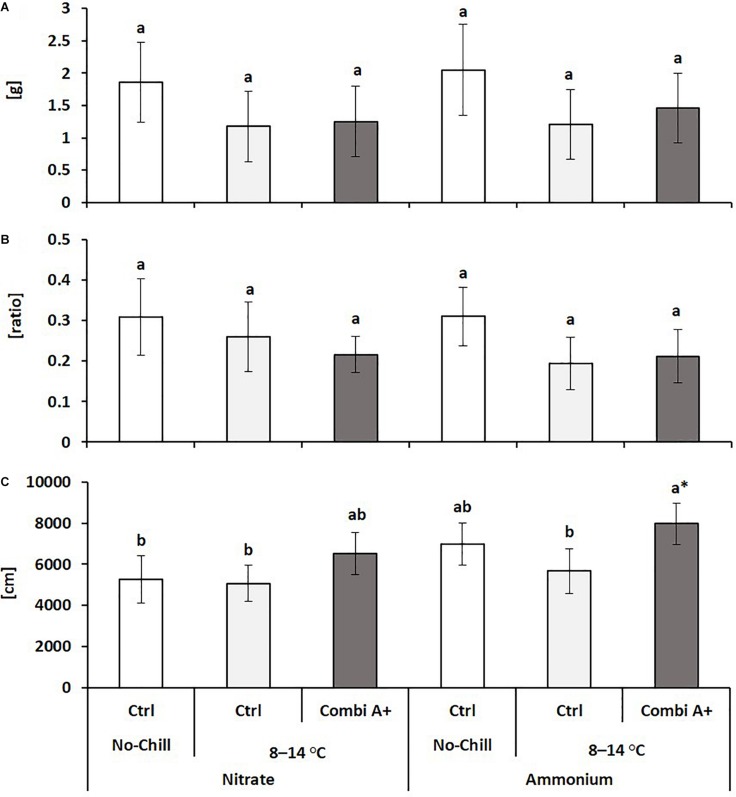
Root dry weight **(A)**, root/shoot biomass ratio **(B)**, and total root length **(C)** of maize plants exposed to a 2-weeks period of reduced root zone temperature (RZT, 8–14°C) on silty clay loam soil, pH 6.9. Un-cooled control: (No-Cold Ctrl) and low RZT variants (8–14°C) with (CombiA^+^) and without (Ctrl) PGPM inoculation under nitrate or stabilized ammonium fertilization. Data represent the means and SD of five replicates. Different letters indicate significant differences (Tukey-Test, *p* < 0.05).

The first experiment addressed the impact of ammonium fertilization versus nitrate supply on the cold-protective performance of the CombiA^+^ consortium. The form of N had no significant effect on the shoot biomass of non-stressed control plants (Table 2). No macro- or micronutrient deficiencies were recorded, irrespective of the N fertilization regime (Supplementary Table S2), while shoot P and Zn accumulation were significantly increased in the ammonium variant (Supplementary Table S2), associated with a decline in rhizosphere pH by 0.6 units as compared with nitrate fertilization (Table 2). The two weeks cold-stress period decreased shoot biomass production of the plants with nitrate supply by 25% (Table 2), associated with a 133% increase in oxidative leaf damage, indicated by chlorosis, necrosis, and stress-induced anthocyanin formation (Figures 1, 2).

**TABLE 2:** Shoot dry weight (DW), oxidative leaf damage (number of damaged leaves plant^–1^), Zn and Mn shoot concentrations and rhizosphere pH of maize plants exposed to a 2-weeks period of reduced root zone temperature on silty clay loam soil, pH 6.9.

N-Form	Stress factor	Treatment	Shoot DW[g]	Oxidative leaf damage [Leaf number]	Zn [μg g^–1^DW]	Mn [μg g^–1^DW]	pH Mean value (CaCl_2_)	pH Δ (Rhizo-Bulk soil)
Nitrate	No-Cold	Ctrl	6.0 ± 0.6 a	2.4 ± 0.54 c	46.0 ± 0.8 b	50.5 ± 1.9 b	7.3 ± 0.02 a	+0.4
	8–14°C	Ctrl	4.5 ± 0.5 b	5.6 ± 0.89 a	24.4 ± 3.9 c	34.8 ± 5.9 c	7.2 ± 0.03 b	+0.3
		Combi A^+^	5.8 ± 0.7 a	3.4 ± 0.554 b	47.8 ± 5.6 b	48.0 ± 1.3 b	7.0 ± 0.05 c	+0.1
Ammonium	No-Cold	Ctrl	6.6 ± 0.2 a	1.6 ± 0.53 c	59.0 ± 2.0 a	51.0 ± 2.6 b	6.7 ± 0.02 d	−0.2
	8–14°C	Ctrl	6.2 ± 0.4 a	3.6 ± 0.52 c	59.4 ± 3.8 a	53.7 ± 1.3 ab	6.7 ± 0.02 d	−0.2
		Combi A^+^	6.9 ± 1.1 a	2.4 ± 0.53 bc	57.6 ± 5.9 a	52.9 ± 3.4 ab	6.6 ± 0.03 e	−0.3

*Un-cooled control: (No-Cold Ctrl) and low RZT variants (8–14°C) with (CombiA^+^) and without (Ctrl) PGPM inoculation under nitrate or stabilized ammonium fertilization. Data represent the means and SD of five replicates. In each row, different letters indicate significant differences (Tukey-Test, p < 0.05).*

Compared with nitrate fertilization, oxidative leaf damage was significantly lower in the ammonium variant and there was no significant decline in shoot biomass (Table 3). The Zn and Mn-nutritional status in cold-stressed plants with nitrate supply dropped close to the deficiency threshold but remained in the sufficiency range in combination with ammonium fertilization (Table 2 and Supplementary Table S2), associated with a lower rhizosphere pH. Nevertheless, ammonium-induced rhizosphere acidification had no effect on the P-nutritional status, which declined below the deficiency threshold in the cold-stressed plants (Supplementary Table S2).

**TABLE 3:** Tissue concentrations of sugars, proline, total phenolics and total antioxidants in maize plants exposed to a 2-weeks period of reduced root zone temperature on silty clay loam soil, pH 6.9.

			Shoot				Root
N-Form	Stress factor	Treatment	Sugar [mg g^–1^ FW]	Proline [mg g^–1^ FW]	Phenolics [mg g^–1^ FW]	Total antioxidants [%]	Total antioxidants [%]
Nitrate	No-Cold	Ctrl	1.7 ± 0.2 d*	0.3 ± 0.04 d*	3.1 ± 0.1 d*	52.5 ± 0.94 d*	39.0 ± 2.5 c*
	8–14°C	Ctrl	2.3 ± 0.2 c	0.5 ± 0.05 c	3.9 ± 0.1 c	67.1 ± 1.89 c	25.7 ± 3.6 d
		Combi A^–^	3.2 ± 0.4 b*	0.7 ± 0.04 b*	4.2 ± 0.4 c	91.4 ± 1.40 b*	62.3 ± 4.7 b*
		Combi A^+^	2.9 ± 0.6 c	0.7 ± 0.03 b*	4.6 ± 0.2 bc*	85.7 ± 6.93 b*	69.6 ± 5.4 ab*
Ammonium	No-Cold	Ctrl	2.3 ± 0.2 c	0.2 ± 0.03 d*	3.9 ± 0.1 c*	58.0 ± 3.80 d*	39.4 ± 2.7 c
	8–14°C	Ctrl	2.5 ± 0.2 c	0.6 ± 0.03 c	4.9 ± 0.1 b	77.1 ± 3.63 bc	29.0 ± 4.0 cd
		Combi A^–^	3.8 ± 0.1 a*	1.0 ± 0.04 a*	5.5 ± 0.5 ab	97.1 ± 1.97 a*	71.5 ± 5.3 b*
		Combi A^+^	3.6 ± 0.2 a*	0.9 ± 0.04 a*	5.6 ± 0.4 a*	98.9 ± 5.92 ab*	87.1 ± 5.2 a*

Un-cooled control: (No-Cold Ctrl) and low RZT variants (8–14°C) with (CombiA^–^; CombiA^+^) and without (Ctrl) PGPM inoculation under nitrate or stabilized ammonium fertilization. CombiA^–^ formulation without Zn/Mn; CombiA^+^ formulation with Zn/Mn. Data represent the means and SD of five replicates. In each row, different letters indicate significant differences (Tukey-Test, p < 0.05). *significant in pairwise comparisons with RTZ untreated Ctrl (t-Test, p < 0.05).

The application of the microbial consortium product CombiA^+^ significantly increased shoot biomass production in the cold-stressed nitrate variant with the same trend in combination with ammonium supply (Table 2), which additionally increased total root length (Figure 2). Under both N form regimes, CombiA^+^ application significantly reduced cold stress-induced oxidative leaf damage but only in the ammonium variant, the level of leaf damage was not significantly different from the non-stressed control (Table 2) and the P status reached the sufficiency range (Supplementary Table S2). No significant differences were recorded for root biomass and the root/shoot biomass ratio (Figure 2).

### Synergistic Effects of N-Form Supply, Micronutrient Supplementation and PGPM Inoculants Adaptive Cold-Stress Responses in Maize

A second experiment was conducted to dissect the individual contributions of ammonium fertilization, Zn/Mn supplementation and CombiA application to the cold-protective effect at the physiological and molecular level. Micronutrient effects were identified by comparison of MCP inoculant formulations with (CombiA^+^) and without additions of Zn/Mn (CombiA^–^).

#### Plant Growth and MCP Root Colonization

**FIGURE 3:**
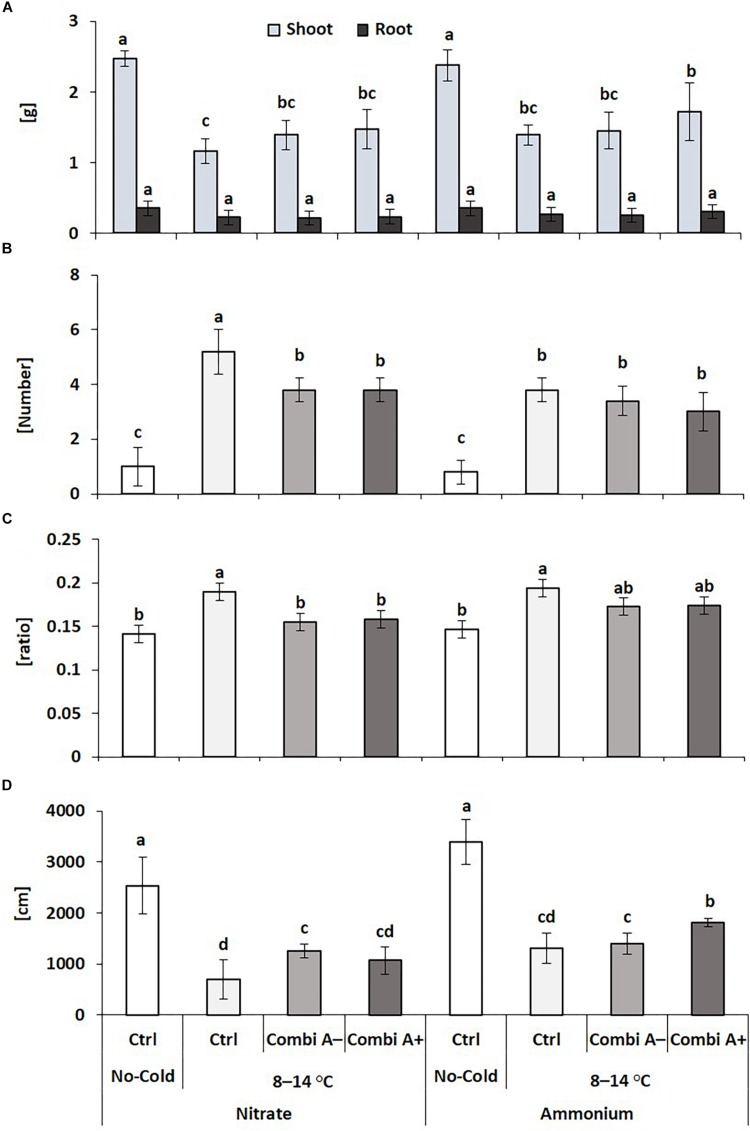
Shoot and root dry weight **(A)**, oxidative leaf damage **(B)** root/shoot biomass ratio **(C)**, and root length **(D)** of maize plants exposed to a 2-weeks period of reduced root zone temperature (RZT, 8–14°C) on silty clay loam soil, pH 6.9. Un-cooled control: (No-Cold Ctrl) and low RZT variants including untreated control (Ctrl), Combi A^–^ (without Zn/Mn) and Combi A^+^ (containing Zn/Mn) under nitrate or ammonium fertilization. Means and SD of five replicates. Different letters: significant differences (Tukey-Test, *p* < 0.05).

Superior cold-protective performance by combined application of stabilized ammonium, Zn/Mn supply, and the PGPM consortium as compared with nitrate fertilization was confirmed also in the second experiment. This was reflected in the highest shoot biomass production (+48%), increased root length (+161%) and the lowest level of cold-stress induced oxidative leaf damage (-42%). Ammonium fertilization and particularly CombiA^+^ application reverted cold-stress induced Zn limitation of the host plants, while root biomass remained unaffected (Figure 3).

**FIGURE 4:**
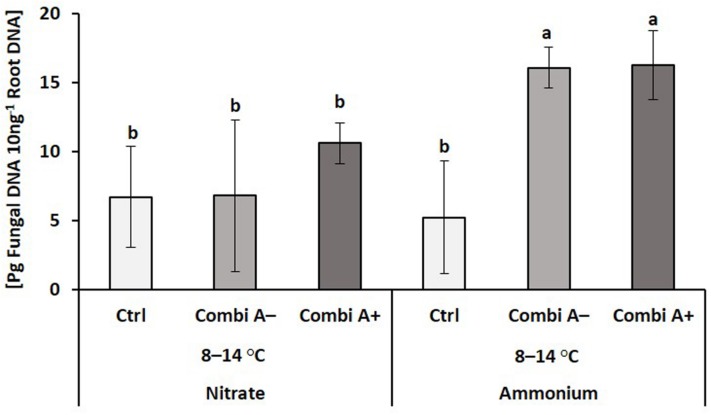
Root colonization with *Trichoderma harzianum* OMG16 of maize plants exposed to a 2-weeks period of reduced root zone temperature on silty clay loam soil, pH 6.9. Un-cooled control: (No-Cold Ctrl) and low RZT variants (8–14°C) with (CombiA^–^; CombiA^+^) and without (Ctrl) PGPM inoculation under nitrate or stabilized ammonium fertilization. CombiA^–^ formulation without Zn/Mn; CombiA^+^ formulation with Zn/Mn. Data represent the means and SD of five replicates. Different letters indicate significant differences (Tukey-Test, *p* < 0.05).

A strain-specific primer was available for *Trichoderma harzianum* OMG16 in the CombiA formulation. This enabled rhizosphere tracing to evaluate the root colonization efficiency of the inoculant. Traces of *T. harzianum* OMG16 DNA were detectable also in the root samples of non-inoculated controls. A significant increase in OMG16 root colonization was recorded exclusively in CombiA-inoculated roots of maize plants with ammonium fertilization and was not affected by additional Zn/Mn supplementation (Figure 4).

#### Accumulation of Antioxidants and Cryoprotective Solutes

Cold stress increased the shoot concentration of proline with cryo-protective an anti-oxidative functions (Szabados and Savouré, 2010) by 67% under nitrate supply and by 200% in the ammonium variant. A significant increase in soluble sugar concentrations was recorded only in combination with nitrate fertilization. Finally, the highest shoot concentrations of proline and soluble sugars accumulated in cold-stressed maize plants with CombiA inoculation and ammonium supply. This could be attributed to the presence of the MCP inoculant since additional Zn/Mn supplementation had no additional effects (Table 3).

Total phenolics and antioxidants increased in the shoot tissue of cold stressed maize plants particularly in combination with ammonium fertilization, while antioxidants in roots rather declined. Again, CombiA inoculation combined with ammonium supply resulted in the highest accumulation of phenolics and total antioxidants, both, in shoot and root tissues. Additional Zn/Mn supplementation further increased the root concentrations of antioxidants (Table 3).

#### Enzymatic ROS Detoxification

**FIGURE 5:**
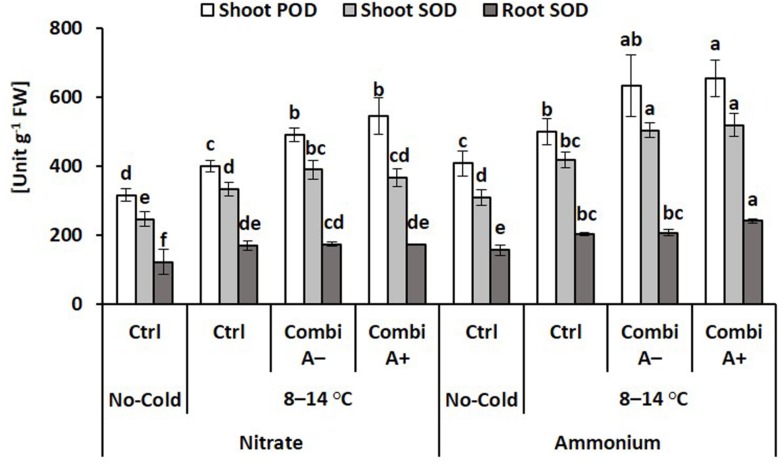
Activities of peroxidase (POD) and superoxide dismutase (SOD) in maize plants exposed to a 2-weeks period of reduced root zone temperature on silty clay loam soil, pH 6.9. Un-cooled control: (No-Cold Ctrl) and low RZT variants (8–14°C) with (CombiA^–^; CombiA^+^) and without (Ctrl) PGPM inoculation under nitrate or stabilized ammonium fertilization. CombiA^–^ formulation without Zn/Mn; CombiA^+^ formulation with Zn/Mn. Bars represent the means and SD of five replicates. For each enzyme, different letters indicate significant differences (Tukey-Test, *p* < 0.05).

Activities of superoxide dismutase and peroxidase were determined as key enzymes involved in the detoxification of cold-stress-induced production of reactive oxygen species. Accordingly, the lowest SOD and POD activities were recorded in the non-stressed controls but with higher values in the ammonium variants. Cold stress further increased SOD and POD in root and shoot tissues with higher levels in plants with ammonium fertilization as compared with nitrate supply. After all, the highest activities were found after CombiA inoculation in combination with ammonium supply. This effect could be mainly attributed to the presence of the MCP inoculant with a small additional impact of Zn/Mn supplementation (Figure 5).

#### Interactions With Hormonal Homeostasis

**FIGURE 6:**
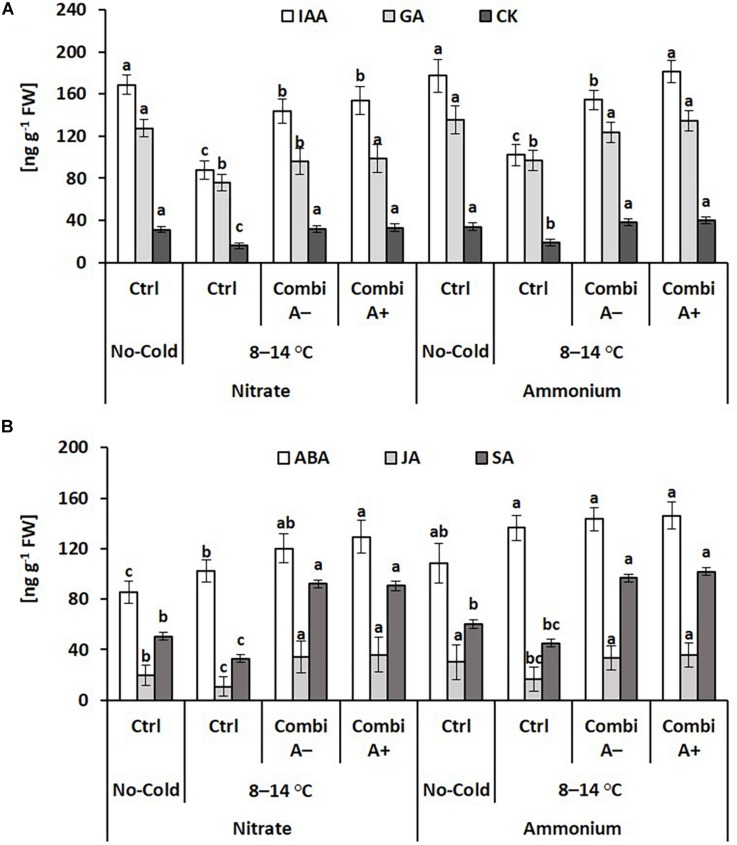
Endogenous concentrations of growth **(A)**, and stress-related **(B)** phytohormones in the shoot tissue of maize plants exposed to a 2-weeks period of reduced root zone temperature on silty clay loam soil, pH 6.9. Un-cooled control: (No-Cold Ctrl) and low RZT variants (8–14°C) with (CombiA^–^; CombiA^+^) and without (Ctrl) PGPM inoculation under nitrate or stabilized ammonium fertilization. CombiA^–^ formulation without Zn/Mn; CombiA^+^ formulation with Zn/Mn. IAA = indole acetic acid: GA = gibberellic acid; CK = cytokinins (zeatin); ABA = abscisic acid; JA = jasmonic acid; SA = salicylic acid. Bars represent the means and SD of five replicates. For each hormone, different letters indicate significant differences (Tukey-Test, *p* < 0.05).

The form of N supply had no effects on the shoot concentrations of the growth hormones indole acetic acid (IAA), gibberellic acid (GA), and zeatin (CK) in non-stressed controlled plants, while the concentrations declined in the cold stress variants without significant differences between plants with nitrate or ammonium supply. The negative cold stress effect on shoot concentrations of IAA, GA, and CK was reverted by CombiA inoculation and more strongly expressed for the GA concentrations in plants with ammonium fertilization as compared with nitrate supply. Additional Zn/Mn supplementation further increased the IAA concentrations in the ammonium variants (Figure 6).

Ammonium fertilization increased the concentrations of the stress hormones abscisic acid (ABA) and jasmonic acid (JA) even in the shoot tissue of non-stressed control plants. Cold stress further increased the ABA levels particularly in the ammonium variant, while the concentrations of JA and salicylic acid (SA) declined. By contrast, JA and SA concentrations increased after CombiA inoculation while ABA increased only in the nitrate variant but not with ammonium supply. Additional effects of Zn/Mn supplementation in the CombiA^+^ variants were not detectable (Figure 6).

**FIGURE 7:**
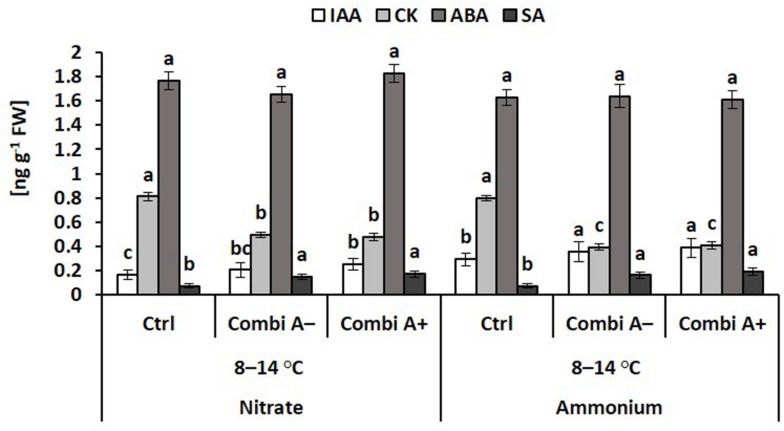
Endogenous concentrations of phytohormones in the root tissue of maize plants exposed to a 2-weeks period of reduced root zone temperature on silty clay loam soil, pH 6.9. Low RZT variants (8–14°C) with (CombiA^–^; CombiA^+^) and without (Ctrl) PGPM inoculation under nitrate or stabilized ammonium fertilization. CombiA^–^ formulation without Zn/Mn; CombiA^+^ formulation with Zn/Mn. IAA = indole acetic acid; CK = cytokinins (zeatin); ABA = abscisic acid; SA = salicylic acid. Data represent the means and SD of five replicates. For each hormone, different letters indicate significant differences (Tukey-Test, *p* < 0.05).

In the roots of cold stressed plants, ammonium supply and particularly the combination with CombiA inoculation increased the IAA tissue concentrations by 75% and 131%, respectively (Figure 7), as similarly recorded also for the shoot tissue (Figure 6). Ammonium fertilization had no effect on the level of root cytokinins (zeatin) but root CK concentrations declined in response to CombiA application, contrary to the opposite effect, recorded in the shoot tissue (Figure 6). The decline in CK concentrations was particularly expressed in the variants with ammonium supply (-50%). CombiA also increased SA concentrations in the root tissue without additional effects by Zn/Mn supplementation (Figure 7). No treatment differences were detectable for root ABA concentrations. Jasmonic acid (JA) ranged below the detection limit.

**FIGURE 8:**
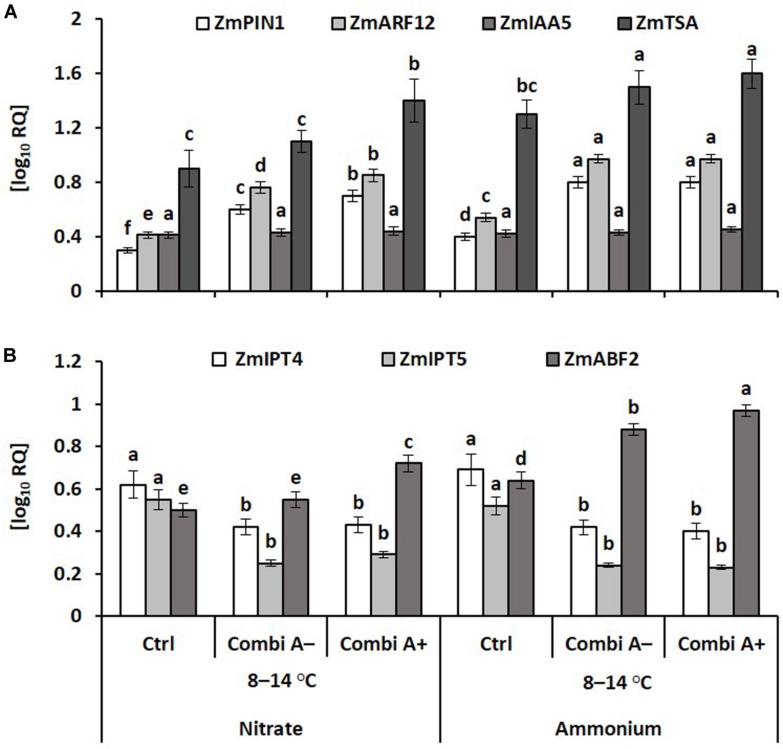
Hormone-related transcript abundances of root tissue in maize plants exposed to a 2-weeks period of reduced root zone temperature (RZT, 8–14°C) on silty loam soil, pH 6.9. Low RZT variants including untreated control (Ctrl), Combi A^–^ (formulation without Zn/Mn) and Combi A^+^ (formulation with Zn/Mn) under nitrate or ammonium fertilization. Means and SD of five replicates. For each gene, different letters: significant differences (Tukey-Test, *p* < 0.05). **(A)** PIN1: PINFORMED1, ARF12: Auxin response factor12, IAA5: Aux/IAA-transcription factor5, TSA: tryptophan synthase, **(B)** IPT4,5: Isopentenyl transferases 4 and 5, ABF2: Abscisic acid-binding factor2.

In accordance with the increased IAA concentrations in the root tissue (Figure 7), expression of the tryptophan synthase gene (*ZmTSA*), involved in the biosynthesis of IAA and other indole compounds (Mano and Nemoto, 2012), was enhanced in response to ammonium fertilization and further increased in combination with CombiA inoculation. This was associated with a correspondingly increased expression of genes encoding for the auxin transporter *ZmPIN1* (Li et al., 2018) and the auxin response factor 12 (*ZmAFR12*) involved in IAA perception (Figure 8A). The *ZmAuxIAA5* gene was selected as a well-studied member of the auxin early response genes, found to be rapidly up-regulated by external auxin supply (Park and Hasenstein, 2016), to test responses to potential IAA production of the inoculants but in this case, no significant treatment differences were detectable (Figure 8A). Declining cytokinin concentrations recorded in the root tissue of CombiA-inoculated plants (Figure 7) were reflected in decreased expression of the genes encoding the isopentenyl transferases 4,5 (*ZmIPT4,5*) involved in cytokinin biosynthesis (Figure 8B). Ammonium fertilization and particularly the combination with CombiA^+^ inoculation increased gene expression of the ABA-responsive ABA-binding factor2; ABF2 (Figure 8B).

## Discussion

### Cold Protective Effects Induced by the Form of N Supply

In both experiments, a certain cold protective effect of stabilized ammonium supply compared with nitrate fertilization was indicated by a 27–36% decline of oxidative leaf damage (chlorosis, necrosis; Figure 1), detectable at the end of the 2-weeks cold stress period (Table 2 and Figure 3). Ammonium fertilization counteracted cold-stress induced zinc and manganese deficiencies, which dropped to critical levels (Campbell and Plank, 2013) in the cold-stressed plants with nitrate supply (Table 2 and Supplementary Table S2). Hence, micronutrient deficiencies (Zn, Mn, Fe) have been characterized as growth-limiting factors for cold-stressed maize plants also in previous studies, reverted by micronutrient supplementation via seed priming (Imran et al., 2013), seed dressing or fertigation (Bradacova et al., 2016; Moradtalab et al., 2018) prior to the onset of the stress period. Under ammonium fertilization, increased shoot concentrations of Zn and Mn were related to the well-documented ammonium-induced rhizosphere acidification (Marschner and Römheld, 1983; Neumann and Römheld, 2002) by 0.6 pH units compared with nitrate supply (Table 2), which apparently increased Zn and Mn solubility in the rhizosphere. However, the rhizosphere acidification effect was obviously not sufficient to mobilize significant amounts of P, since the P status remained in the deficiency range (Supplementary Table S2).

Components of both, enzymatic and non-enzymatic ROS detoxification are particularly dependent on sufficient micronutrient supplying (Cakmak, 2000; Datnoff et al., 2007), providing enzymatic co-factors for superoxide dismutases (Zn, Mn, Fe, Cu), peroxidases (Fe) and enzymes involved in biosynthesis of phenolics with antioxidative potential (Mn, Cu). Consequently, Zn/Mn supplementation by seed dressing increased superoxide dismutase (SOD) activity, accumulation of phenolics and antioxidants associated with a decline in ROS accumulation and reduced oxidative leaf damage in cold-stressed maize plants (Bradacova et al., 2016; Moradtalab et al., 2018). Similarly, in this study, an improved Zn/Mn status in response to ammonium-induced rhizosphere acidification (Table 2) may be related with increased activities of superoxide dismutases (+ 25%) and peroxidases (+ 25%; Figure 5) and increased Mn-dependent shoot accumulation of phenolics (+ 26%; Table 3), while oxidative leaf damage declined by 27% (Figure 3). This was associated with increased shoot concentrations of abscisic acid (ABA, Figure 6) as a central regulator of cold stress adaptations in plants (Szalai et al., 2011; Eremina et al., 2016). Direct links between ABA and enzymatic ROS detoxification in cold-stressed plants have been reported by Kumar et al. (2008), Szalai et al. (2011), Li and Zhang (2012), and Moradtalab et al. (2018), while Peuke et al. (1994) reported ammonium-induced stimulation in root to shoot translocation of ABA in *Ricinus* seedlings Accordingly, the cold-protective effect of ammonium fertilization observed in this study may be related with a stimulatory effect on ABA accumulation in the shoot tissue, which promoted the expression of enzymatic (SOD, POD, Figure 6) and non-enzymatic (phenolics, Table 3) ROS detoxification. Similarly, ammonium-induced induction of ABA accumulation and a relationship with improved oxidative stress defense was reported also in response to other abiotic stress factors such as drought and salinity (Hessini et al., 2013; Ding et al., 2016).

In the root tissue of cold-stressed plants, ammonium fertilization significantly increased the IAA concentration by 41% as compared with nitrate supply (Figure 7), with a similar trend for shoot IAA, which was not detectable in the absence of cold stress. In our study, this was related to a significantly increased expression of the *ZmPIN1a* gene (Figure 6A), encoding an auxin efflux carrier with functions in the lateral root formation in maize (Li et al., 2018). Increased gene expression was recorded also for the auxin response factor 12 (*ZmAFR12*) involved in IAA perception (Figure 6A) and upregulated in cold-stressed maize plants (Sobkowiak et al., 2014). Thus, a trend for increased root length development in the ammonium-treated plants was recorded in both experiments, although this difference was not statistically significant (Figure 3). Excessive production of ROS can promote the oxidative degradation of IAA. and resulted in a 50% reduction of IAA contents in Zn-deficient *Phaseolus vulgaris*, which was reverted by Zn fertilization (Cakmak et al., 1989), promoting enzymatic ROS detoxification (Cakmak, 2000). Similarly, a ROS-protective effect of higher SOD activities recorded in the root tissue of ammonium-treated plants (Figure 5) may counteract oxidative IAA degradation and provide an explanation for greater root IAA levels in cold-affected maize over nitrate-treated plants (Figure 7).

As an additional beneficial effect of ammonium fertilization, root colonization by the CombiA-PGPM strain *Trichoderma harzianum* OMG16 was increased in comparison with plants supplied with nitrate fertilizer (Figure 4). The reasons for this preference are not entirely clear but recently Mpanga et al. (2019a) found ammonium-induced promotion of root hair development in P-deficient maize plants, also identified as limiting nutrients in this study (Supplementary Table S2). This may provide additional infection sites, since preferential colonization of root hairs has been reported for various strains of *Trichoderma harzianum*, including T22 and OMG16 (Harman, 2000; Mpanga et al., 2019a). Additionally, the various cold stress-protective effects, induced by ammonium fertilization as described above, may improve the rhizosphere establishment of the inoculants by strengthening the host plant. Similarly, improved performance of a wide range of bacterial and fungal PGPMs, including single strain inoculants and microbial consortia in combination with stabilized ammonium fertilization, has been documented in various pot and field experiments under conditions of P limitation (Bradáčová et al., 2019a, b; Mpanga et al., 2019a, b).

### Cold-Protective Effects of the CombiA Inoculation as Related to Zn and Mn Supplementation

For both forms of nitrogen fertilization, the inoculation with CombiA induced cold-protective effects in maize plants, which were still detectable after a two-weeks recovery period at soil temperatures ≥20°C. This may indicate not only direct stress mitigation, as indicated e.g., by reduced oxidative leaf damage recorded at the end of the 2-weeks cold stress period (Table 2 and Figure 3) but also the induction of longer-lasting stress priming effects.

The most intense expression of cold protection in terms of increased shoot biomass production, reduced oxidative leaf damage, and stimulation of root growth, was recorded for the ammonium-CombiA^+^ combination (Table 2 and Figures 2, 3). The effects were detectable in both experiments conducted under controlled root zone temperatures (RZT), although shoot biomass production was different, probably due to differences in ambient air temperature at the greenhouse (Supplementary Figure S1).

The two-weeks cold stress treatments with 8–14°C RZT reduced the shoot dry matter production by 25–52% when nitrate was the N source (Table 2 and Figure 3). This was associated with a significant decline in the shoot concentrations of the growth hormones IAA, GA, and CK (Zeatin) by 48, 41 and 49%, respectively (Figure 6), as previously reported also by Moradtalab et al. (2018). Reduction of shoot growth is regarded as a component of cold stress adaptations, which is actively regulated by a reduction of bioactive growth-promoting gibberellic acid (GA) levels, leading to an increased abundance of nuclear DELLA-protein growth repressors via a signaling pathway involving CBF/DREB1 transcription factors (Miura and Furumoto, 2013; Eremina et al., 2016). However, the decline of GA and IAA in cold stressed plants have been also related to cold-induced Zn-limitation (Moradtalab et al., 2018) since reduced GA and IAA levels are characteristic for Zn-deficient plants (Suge et al., 1986; Sekimoto et al., 1997; Cakmak et al., 1989). More recently, it was shown that various steps of GA biosynthesis depend on the presence of IAA (Ross et al., 2000) and Zn limitation promotes oxidative IAA degradation and impairs auxin transport (Cakmak et al., 1989; Shibasaki et al., 2009).

Interestingly, two weeks after recovery from the cold stress treatment, CombiA inoculation particularly in combination with ammonium fertilization, restored the concentrations of IAA, GA, and CK to the levels characteristic for non-stressed plants (Table 4). This was associated with the lowest level of oxidative leaf damage, increased shoot biomass production (Table 2, Figures 2, 3), increased enzymatic (POD, SOD) and non-enzymatic (total antioxidants, phenolics, proline) ROS defense and accumulation of cryoprotectants (Table 4 and Figure 5), indicating an improved recovery from the cold stress treatment. Strengthening of ROS detoxification in the shoot tissue was not related to Zn and Mn supplementation by the CombiA^+^ treatment since the same effect was observed also for CombiA^–^ inoculation without additional micronutrient supply (Tables 4, 5). In this case, the improved micronutrient supply, induced by ammonium fertilization (Table 2), was already sufficient to cover the requirements of the systems for ROS detoxification.

**TABLE 4:** The ratio of abscisic acid/cytokinin (ABA/CK) and auxin/cytokinin (IAA/CK) concentrations in the root of maize plants exposed to a 2-weeks period of reduced root zone temperature (RZT, 8–14°C) on silty clay loam soil, pH 6.9.

	8–14°C					
	Nitrate			Ammonium		
Ratio	Ctrl	Combi A^–^	Combi A^+^	Ctrl	Combi A^–^	Combi A^+^
ABA/CK	2.18 c	3.33 b	3.81 b	2.04 c	4.14 a	3.96 a
IAA/CK	0.21 c	0.42 b	0.53 b	0.37 c	0.91 a	0.96 a

*Low RZT variants including untreated control (Ctrl), Combi A^–^ (without Zn/Mn) and Combi A^+^ (containing Zn/Mn) under nitrate or ammonium fertilization. Means of five replicates. In each row different letters: significant differences (Tukey-Test, p < 0.05).*

**TABLE 5:** Relative changes (%) of phenotypic and physiological responses in maize plants induced by stabilized ammonium fertilization (Ctrl ammonium), ammonium fertilization + PGPM inoculation (Ammonium + CombiA^–^) and ammonium fertilization + PGPM inoculation + Zn/Mn supplementation (Ammonium + CombiA^+^) after recovery (14 days) from two weeks exposure to low root zone temperatures (8–14°C) over plants supplied with N in the nitrate form.

Tissue	Factor	Ctrl (Ammonium)	Ammonium + Combi A^–^	Ammonium + Combi A^+^
Root	Length	n.s	+101	+161
	PGPM Colonization	n.s	+140	+143
	**ROS Defense**			
	Antioxidants	n.s	+178	+239
	SOD	+20	+22	+42
	**Hormonal Effects**			
	IAA	+75	+112	+131
	CK	n.s	−51	−50
	ABA	n.s	n.s	n.s
	SA	n.s	+123	+162
	ZmPIN1	+33	+167	+167
	ZmARF12	+32	+137	+137
	ZmIAA5	n.s	n.s	n.s
	ZmTSA	n.s	+67	+78
	ZmIPT4	n.s	−32	−35
	ZmIPT5	n.s	−56	−58
	ZmABF2	+28	+76	+94
Shoot	Biomass	n.s	n.s	+48
	Oxidative leaf damage	−27	−35	−42
	**ROS Defense**			
	SOD	+25	+52	+56
	POD	+25	+59	+64
	Phenolics	+26	+41	+44
	Antioxidants	n.s	+45	+47
	**Cryoprotectants**			
	Proline	n.s	+100	+80
	Sugar	n.s	+65	+57
	**Hormonal Effects**			
	IAA	n.s	+76	+106
	GA	n.s	+63	+78
	CK	n.s	+141	+153
	ABA	+33	+40	+43
	JA	+55	+208	+231
	SA	+38	+195	+211

*SOD: Superoxide dismutase, POD: Peroxidase, IAA: Auxin, CK: Cytokinin, GA: Gibberellic acid, ABA: Abscisic acid, JA: Jasmonic acid, SA: Salicylic acid, PIN1: PINFORMED1, ARF12: Auxin response factor12, IAA5: Aux/IAA-transcription factor5, TSA: tryptophane synthase, IPT4,5: Isopentenyl transferases4,5, ABF2: Abscisic acid-binding factor2, n.s: not significant.*

Also increased ABA production with the potential to trigger ROS defense was not detectable in CombiA treatments but was characteristic for sole ammonium supply (Figure 6). By contrast, CombiA inoculation was related to increased shoot accumulation of JA and SA (Figure 6). This points to induction of induced systemic resistance (ISR) via JA and SA signaling pathways, which is well documented for various *Trichoderma* and *Bacillus* strains, with stimulatory effects e.g., on the accumulation of phenolics and POD activity (García-Gutiérrez et al., 2013; Martínez-Medina et al., 2013, 2014, Shahzad et al., 2017). Unfortunately with the currently available data set, it is not possible to unfold the individual contributions of the selected *Trichoderma* and *Bacillus* inoculants to the ISR effect. This would require additional experiments with single-strain inoculations. Although abscisic acid is considered as a central regulator of cold stress responses in plants, it seems to regulate the adaptive expression of cold- related genes in cross talks involving also SA and JA (Szalai et al., 2011; Eremina et al., 2016). This may also explain the improved cold acclimation by CombiA inoculation via ISR-induced production of JA and SA (Table 5).

The only superior cold-protective feature related to the increased Zn/Mn supply provided by CombiA^+^ in combination with ammonium fertilization recorded in this study was the increased accumulation of antioxidants in the root tissue (Table 3), which promoted root elongation (Figures 2, 3). Since at the same time, root biomass production was not significantly affected, obviously fine root production was stimulated, characterized by a higher root length with the same root biomass after CombiA^+^ application as compared to the CombiA^–^ variant (Figures 2, 3). This may indicate a protective effect against oxidative auxin degradation leading to root growth inhibition (Cakmak et al., 1989, Moradtalab et al., 2018). Generally, CombiA inoculation increased IAA concentrations not only in the shoot but also in the root tissue, associated with a decline in root CK (Figure 5). This was related to increased expression of auxin-responsive genes involved in IAA biosynthesis (*ZmTSA*), transport (*ZmPIN1A*) and IAA signal perception (*ZmARF12*), while the expression of genes involved in CK biosynthesis (*ZmIPT4* and *5*) declined (Figure 6A). By contrast, the expression of the *AuxIAA5* gene (*ZmIAA5*), reported to be rapidly activated by exogenous IAA supply (Park and Hasenstein, 2016), was not changed by CombiA inoculation. This finding suggests that CombiA rather acted via signals interacting with internal IAA homeostasis of the host plant and not via microbial IAA production. Accordingly, Garnica-Vergara et al. (2015) found an auxin-independent activation of *PIN* genes (*PIN1, PIN2, PIN3, PIN7*), associated with increased lateral root formation in *Arabidopsis thaliana* by 6-pentyl-2H-pyran-2-one (6-PP), a major bioactive volatile organic compound (VOC) with potential cross-kingdom signaling functions, emitted by *Trichoderma* spp. Interestingly, increased levels of IAA and declining CK concentrations in the root tissue in response to *Trichoderma* inoculation detected in our study, have been observed also in earlier reports with melon seedlings and cherry rootstocks (Sofo et al., 2011; Martínez-Medina et al., 2014). This strongly suggests that the corresponding hormonal changes induced by CombiA inoculation (Figures 5, 6) are likely caused by the *T. harzianum* OMG16 strain in the inoculum, associated with preferential root colonization in combination with ammonium fertilization (Figure 4). Similar effects on root growth and plant IAA homeostasis have been reported also for certain N-acyl homoserine lactones secreted by various rhizosphere bacteria for intercellular communication (quorum sensing; Hartmann et al., 2014).

Auxin production is considered as a central feature of *Bacillus* strains leading to root growth promotion via external supplementation of IAA by the inoculant (Borriss, 2015). Hence, IAA production has been reported for many *Bacillus* species, such as *B. subtilis* (Hashem et al., 2019), *B. megaterium* (Marulanda et al., 2009), *B. licheniformis* (Singh and Jha, 2016) and *B. velezensis* (Mpanga et al., 2019a), where IAA production was even stimulated in the presence of ammonium fertilizers. On the other hand, inactivation of genes responsible for bacterial tryptophan synthesis inhibited IAA formation and plant growth promotion (Idris et al., 2007). However, in our study, no upregulation of the *ZmIAA5* gene, activated by external IAA supplying was detectable (Figure 8A), suggesting that external IAA supplementation by the bacterial inoculants was not involved in the observed root growth increase, at least under the investigated cold stress conditions.

Declining CK concentrations induced by CombiA inoculation also had important consequences for the hormonal balances in the root tissue, known to be even more important for the hormonal regulation of physiological processes than the absolute concentrations of individual phytohormones (Nordström et al., 2004; Mueller and Leyser, 2011). The decline in root CK increased the IAA/CK ratio by factor 3 and doubled the ABA/CK ratio in plants with CombiA inoculation and supplying of N as ammonium (Table 4).

Since CKs are acting as potent hormonal antagonists of IAA, e.g., by inhibiting polar IAA transport mediated by PIN transporters (Fukaki and Tasaka, 2009), the increased IAA/CK ratio in CombiA treated plants (Table 4), may result in an improved shoot to root allocation of IAA, increased IAA activity with subsequent stimulation of root growth (Figures 2, 3). Similarly, antagonistic effects of CK on ABA-mediated responses have been reported (Wilkinson et al., 2012; Pavlů et al., 2018). Therefore, the increased ABA/CK ratio may promote ABA-induced induction of cold-adaptations in the root tissue, although endogenous ABA concentrations were not changed (Figure 8), as similarly reported also for cold acclimation in durum wheat (Veselova et al., 2005). This is in line with increased gene expression of the ABA response factor *ZmABF2* particularly in the root tissue of CombiA^+^-inoculated plants with ammonium supply (Figure 6B), known to be upregulated in cold-stressed maize plants (Sobkowiak et al., 2014).

### Contribution of N Forms, Zn, Mn, and Microbial Inoculants to the Cold-Protective Maize Response

**FIGURE 9:**
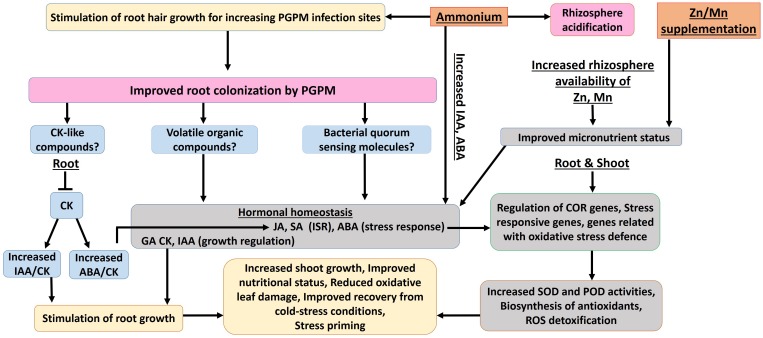
Proposed interactions of stabilized ammonium fertilization, PGPM (CombiA) inoculation and Zn/Mn supplementation contributing to increased cold tolerance during the early growth of maize (for description of details see section 6.2 and 6.3).

In summary, the results of the present study indicate a differential activation and stimulation of adaptive cold-stress responses, induced by the selected fertilization strategies. The various complementary and synergistic effects of ammonium fertilization, CombiA inoculation and ZnMn supplementation on cold stress adaptations in maize are schematically summarized in Figure 9, while Table 4 provides an overview of the relative importance of the selected mitigation strategies (ammonium fertilization, PGPM inoculation, Zn/Mn supplementation) for the expression of cold-protective effects.

#### Effects of Ammonium Fertilization

In accordance with the hypothesis (i), ammonium-dominated fertilization stimulated rhizosphere acidification, which improved the availability and the nutritional status of critical nutrients such as Zn and Mn on the investigated soil with neutral pH (Figure 9), although the effect on P availability was marginal. The improved Zn and Mn-nutritional status, with important functions in oxidative stress defense, moderately increased the enzymatic and non-enzymatic ROS detoxification, counteracted oxidative IAA degradation and oxidative leaf damage (Table 5). Ammonium fertilization was also the major factor contributing to increased ABA concentrations in the shoot tissue (Table 5), as a central regulator of adaptive cold stress responses and stimulated root colonization with the PGPM inoculant CombiA (Figure 9).

#### Effects of PGPM (CombiA) Inoculation

Root growth promotion by stimulation of IAA biosynthesis and reduction of antagonistic cytokinins in the root tissue of the host plant, was a major feature provided by PGPM inoculation with CombiA. Additionally, PGPM inoculation was associated with typical responses of ISR signaling via induction of jasmonic and salicylic acid accumulation even in the shoot tissue and an increase in the ABA/cytokinin ratio in roots. This was related with a further increase in enzymatic (SOD, POD) and non-enzymatic (antioxidants, phenolics, proline) ROS detoxification expressed mainly in the shoot tissue, and consequently a further decline of oxidative leaf damage. The observed effects are in line with the assumptions of the initial hypothesis (iii).

#### Effects of Zn/Mn Supplementation

Partially in line with the hypothesis (ii), the additional supplementation with Zn and Mn mainly contributed to an additional increase of antioxidants and SOD activity in the root tissue. This was associated with increased IAA accumulation, reflecting a reduction of oxidative IAA degradation, which is typically induced under Zn deficiency related with high soil pH and impairment of root activity under cold stress. In consequence, further stimulation of root growth contributed to improved nutrient (P) acquisition, a generally improved plant nutritional status, improved plant performance and induced longer-lasting stress priming effects, still detectable two weeks after recovery from the cold stress treatments.

## Conclusion

The combined use of N as ammonium, Mn, Zn and the Trichoderma/Bacillus inoculant is a suitable strategy to improve the tolerance of maize plants in the early growth stage to cold-stress conditions. This approach could be easily integrated into existing strategies for starter fertilization of maize production systems, such as seedbed fertilization with stabilized ammonium phosphates and micronutrient supplementation in combination with granulated spore formulations of the Trichoderma/Bacillus inoculant. Field performance of the agronomic practice proposed needs further evaluation in field trials, mirroring the already demonstrated effectiveness of single applications of micronutrients and silicon to improve the growth of maize plants (Imran et al., 2013; Moradtalab et al., 2018). Due to overlapping, adaptive plant responses to several abiotic stress factors, and additional biocontrol properties of the inoculants, even a wider spectrum of stress-protective effects might be expected.

## Data Availability Statement

All datasets generated for this study are included in the article/Supplementary Material.

## Author Contributions

NM and GN conceived and designed the experiments. NM and AA conducted the experiments, performed the analyses, and collected the data. GN, UL, FW, BH, and JG provided the facilities for analyses. GN, UL, JG, and FW read and edited the manuscript. All authors approved the final manuscript. NM and AA equally contributed to manuscript writing.

## Conflict of Interest

The authors declare that the research was conducted in the absence of any commercial or financial relationships that could be construed as a potential conflict of interest.
