# Supplementary material for: Synergisms of Microbial Consortia, N Forms, and Micronutrients Alleviate Oxidative Damage and Stimulate Hormonal Cold Stress Adaptations in Maize
Source: Front Plant Sci. 2020 Apr 24;11:396. doi: 10.3389/fpls.2020.00396 (PMC7193188; doi:10.3389/fpls.2020.00396)
Supplement: Supplementary file 1 [file Data_Sheet_1.docx]

**Synergisms of microbial consortia, N forms, and micronutrients alleviate oxidative damage and stimulate hormonal cold stress adaptations in maize**

Narges Moradtalab^1*^, Aneesh Ahmed^1^, Joerg Geistlinger^2^, Frank Walker^3^, Birgit Höglinger^3^, Uwe Ludewig^1^, Günter Neumann^1^

1 Institute of Crop Science (340h), University of Hohenheim, Stuttgart, Germany

2 Institute of Bioanalytical Sciences, Anhalt University of Applied Sciences, Bernburg, Germany

3 Institute of Phytomedicine (360), University of Hohenheim, Stuttgart, Germany

*Corresponding author: Narges Moradtalab, n.moradtalab@uni-hohenheim.de

**9. Supplementary Material**


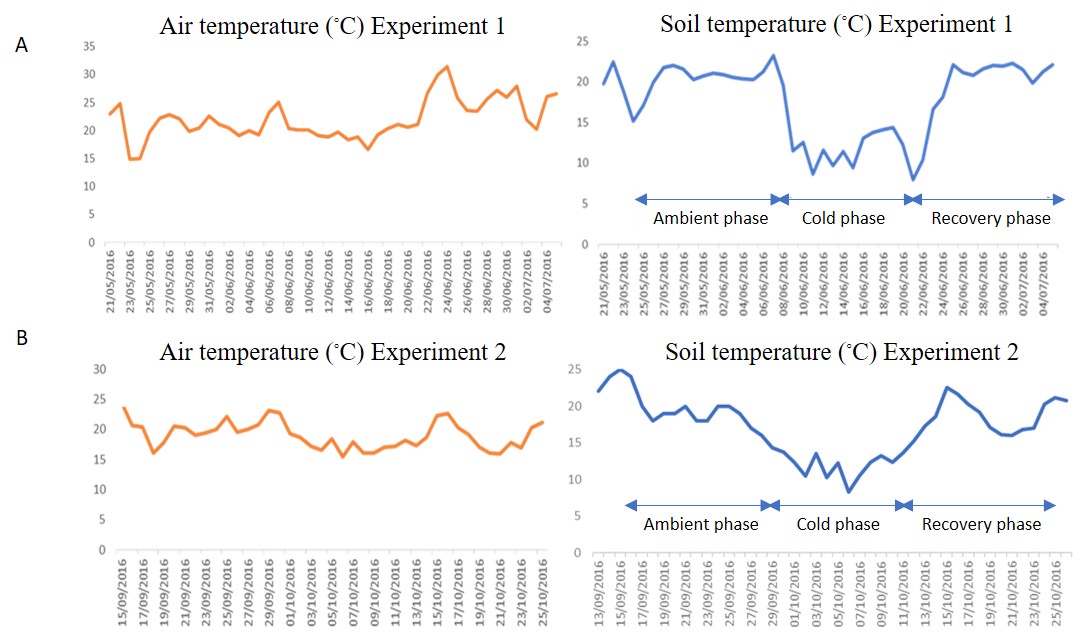


**Figure. S1.** Time courses of ambient air and soil temperature during A) Experiment 1 and B) Experiment 2.

**Table. S1.** Shoot and root dry weight (DW) and cold-stress induced oxidative leaf damage of maize plants exposed to a 2-weeks period of reduced root zone temperature (RZT, 8–14 ^○^C) on a silty clay loam field soil, pH 6.9 with nitrate fertilization.

Uncooled positive control (No-Cold Ctrl) and low RZT variants including (i) a negative untreated control (Ctrl), (ii) Zn/Mn: seed dressing with Zn/Mn, (ii) Zn/Mn + ABI02: *Bacillus atrophaeus* ABI02 combined with Zn/Mn seed dressing, (iii) Zn/Mn +BFOD: *Penecillium* sp. BFOD combined with Zn/Mn seed dressing, and (iv) Combi A^+^: *Trichoderma harzianum OMG16* + Vitabac. Supplemented with Zn/Mn Data represent means and SD of five replicates. Different letters indicate significant differences (Tukey-Test, p < 0.05); *: significant in pairwise comparisons with 8-14°Ctrl. (t-Test, p <0 .05).

| N-Form | Stress | Treatment | Shoot DW [g] | Root DW [g] | Oxidative leaf damage  [leaves plant^-1^] |
| --- | --- | --- | --- | --- | --- |
| Nitrate | No-Cold | Ctrl | 6.0±0.6 a* | 1.9±0.2 a* | 2.4±0.5 c* |
|  | 8–14 ^○^C | Ctrl | 4.5±0.5 c | 1.2±0.2 b | 5.6±0.9 a |
|  |  | Zn/Mn | 5.1±0.5 bc | 1.1±0.2 b | 4.4±1.1 a |
|  |  | Zn/Mn +ABI02 | 5.2±0.3 b | 1.2±0.2 b | 4.6±0.5 a |
|  |  | Zn/Mn +BFOD | 5.8±0.2 ab* | 1.2±0.3 b | 4.4±0.5 ab* |
|  |  | Combi A^+^ | 5.8±0.7 ab* | 1.3±0.2 b | 3.4±0.5 b* |

(i) Seed dressing with Zn/Mn Lebosol® Dünger GmbH, Ermstein, Germany), (ii) Zn/Mn seed dressing + fertigation with 10^9^ spores Kg^-1^ soil DM ABI02, a cold-tolerant *Bacillus atrophaeus* strain (ABITEP, Berlin, Germany), (iii) Zn/Mn seed dressing + fertigation with 10^8^ spores Kg^-1^ soil DM biological fertilizer OD (BFOD), a *Penicillium* sp. formulation (Bayer Crop Science Biologicals GmbH, Malchow, Germany), (iv) Fertigation with 2.5×10^7^ cfu Kg^-1^ soil DM Combi A^+^, a combination product of Zn (13%w(w) + Mn (9%w/w) + *Trichoderma harzianum OMG16* (9 ×10^9^ spores g^-1^) + Vitabac (1 ×10^11^ cfu g^-1^, mixture of *Bacillus licheniformis*, *B. megaterium, B. polymyxa, B. pumilis and B. subtilis,* Bactvita GmbH, Straelen, Germany).

**Table. S2.** A) Mineral concentrations in shoot dry matter (DM) with published deficiency thresholds, and B) total shoot contents of minerals in maize plants exposed to a 2-weeks period of reduced root zone temperature on silty clay loam soil, pH 6.9. Un-cooled control: (No-Cold Ctrl) and low RZT variants (8–14 °C) with (CombiA+) and without (Ctrl) PGPM inoculation under nitrate or stabilized ammonium fertilization. Data represent the means and SD of five replicates. Different letters indicate significant differences (Tukey-Test, p < 0.05).

| **A** |  |  |  |  |  |
| --- | --- | --- | --- | --- | --- |
| N-Form | Stress Factor | Treatment | Ca [mg g^-1^ DM] | Mg [mg g^-1^ DM] | P [mg g^-1^ DM] |
| Nitrate | No-Cold | Ctrl | 4.85 a | 2.24 a | 3.76 a |
|  | 12–14 ^○^C | Ctrl | 3.79 b | 1.85 a | 1.11 c |
|  |  | Combi A^+^ | 5.10 a | 2.28 a | 3.01 b |
| Ammonium | No-Cold | Ctrl | 4.69 a | 2.14 a | 3.79 a |
|  | 12–14 ^○^C | Ctrl | 3.13 b | 1.44 a | 1.43 c |
|  |  | Combi A^+^ | 4.31 ab | 1.96 a | 4.14 a |
| Deficiency threshold* | |  | 2.5 | 1.5 | 3 |
| N-Form | Stress Factor | Treatment | Zn [µg g^-1^ Dm] | Mn [µg g^-1^ DM] | Cu [µg g^-1^ DM] |
| Nitrate | No-Cold | Ctrl | 46.00 a | 50.51 a | 4.82 a |
|  | 12–14 ^○^C | Ctrl | 24.40 b | 34.82 b | 4.03 a |
|  |  | Combi A^+^ | 47.81 a | 48.01 a | 4.16 a |
| Ammonium | No-Cold | Ctrl | 59.01 a | 51.04 a | 4.30 a |
|  | 12–14 ^○^C | Ctrl | 59.42 | 53.72 a | 4.06 a |
|  |  | Combi A^+^ | 57.63 | 52.93 a | 4.88 a |
| Deficiency threshold* | |  | 20 | 20 | 5 |
| **B** |  |  |  |  |  |
| N-Form | Stress Factor | Treatment | Ca [mg Plant^-1^] | Mg [mg Plant^-1^] | P [mg Plant^-1^] |
| Nitrate | No-Cold | Ctrl | 29.10 a | 13.44 a | 22.56 a |
|  | 12–14 ^○^C | Ctrl | 17.06 b | 8.33 b | 5.00 c |
|  |  | Combi A^+^ | 29.58 a | 13.22 a | 17.46 b |
| Ammonium | No-Cold |  | 30.95 a | 14.12 a | 25.01 a |
|  | 12–14 ^○^C |  | 19.41 b | 8.93 b | 8.87 b |
|  |  |  | 29.74 a | 13.52 a | 28.57 a |
| N-Form | Stress Factor | Treatment | Zn [µg Plant^-1^] | Mn [µg Plant^-1^] | Cu [µg Plant^-1^] |
| Nitrate | No-Cold | Ctrl | 276.00 a | 300.06 a | 28.92 a |
|  | 12–14 ^○^C | Ctrl | 109.80 b | 156.69 b | 18.14 b |
|  |  | Combi A^+^ | 277.24 a | 278 .46 a | 24.13a |
| Ammonium | No-Cold | Ctrl | 389.40 a | 336.86 a | 28.38 a |
|  | 12–14 ^○^C | Ctrl | 368.40 a | 333.06 a | 25.17 a |
|  |  | Combi A+ | 397.65 a | 365.22 a | 33.67 a |

* Campbell C. R. and Plank C. O. (2013). Chapter: Reference Sufficiency Ranges — Field Crops/Corn at Early Growth. Campbell C. R. (Ed.). In Reference sufficiency ranges for plant analysis in the southern region of the united states (3rd ed.), southern cooperative series Bulletin #394. p. 122.
